# Structural Analysis of Inhibitor Binding to CAMKK1 Identifies Features Necessary for Design of Specific Inhibitors

**DOI:** 10.1038/s41598-018-33043-4

**Published:** 2018-10-04

**Authors:** André da Silva Santiago, Rafael M. Couñago, Priscila Zonzini Ramos, Paulo H. C. Godoi, Katlin B. Massirer, Opher Gileadi, Jonathan M. Elkins

**Affiliations:** 1 Structural Genomics Consortium, Universidade Estadual de Campinas, Cidade Universitária Zeferino Vaz, Av. Dr. André Tosello, 550, Barão Geraldo, Campinas, SP 13083-886 Brazil; 2 Center for Molecular Biology and Genetic Engineering, CBMEG, University of Campinas, Av Candido Rondon, 400, Barao Geraldo, Campinas, SP 13083-875 Brazil; 3 Structural Genomics Consortium, University of Oxford, Old Road Campus Research Building, Roosevelt Drive, Oxford, OX3 7DQ UK

## Abstract

The calcium/calmodulin-dependent protein kinases (CAMKKs) are upstream activators of CAMK1 and CAMK4 signalling and have important functions in neural development, maintenance and signalling, as well as in other aspects of biology such as Ca^2+^ signalling in the cardiovascular system. To support the development of specific inhibitors of CAMKKs we have determined the crystal structure of CAMKK1 with two ATP-competitive inhibitors. The structures reveal small but exploitable differences between CAMKK1 and CAMKK2, despite the high sequence identity, which could be used in the generation of specific inhibitors. Screening of a kinase inhibitor library revealed molecules that bind potently to CAMKK1. Isothermal titration calorimetry revealed that the most potent inhibitors had binding energies largely dependent on favourable enthalpy. Together, the data provide a foundation for future inhibitor development activities.

## Introduction

The calcium/calmodulin-dependent kinases (CAMKs) are of great interest due to their important functions in calcium signalling and especially in neuronal development^1–3^. The CAMK family is formed by CAMK1, CAMK2, CAMK3 (eEF2 kinase), CAMK4 and the CAMK kinases (CAMKKs)^1,4^. Each member of the CAMK family is composed of a catalytic protein kinase domain followed by an auto-inhibitory sequence, which is partially overlapped with a calmodulin-binding domain (CBD). The auto-inhibitory sequence keeps the CAMKs in an inactive state until activation by calcium/calmodulin (Ca^2+^/CaM). Activation occurs by Ca^2+^/CaM binding to the CBD of a CAMK, which releases the auto-inhibitory sequence from the kinase domain^1,2^.

CAMK1, CAMK4 and CAMKKs have a pivotal role in the cascade responsible for Ca^2+^/CaM signalling. This cascade is known to be crucial for neuronal maturation and axon elongation^4^. The CAMKKs are Ser/Thr kinases and the upstream activator kinases of the CaM-stimulated kinase cascade in which activation of CAMK1 and CAMK4 requires phosphorylation at Thr177 and Thr196, respectively, by CAMKKs. There are two CAMKKs, CAMKK1 (CAMKKα) and CAMKK2 (CAMKKβ)^5–7^. There is extensive data supporting the importance of CAMKKs in neurite development, axon elongation, long term potentiation, and long-term memory (LTM), via activation of cAMP-responsive element binding protein (CREB), which is essential for LTM formation^8,9^. Together the data support the value of investigating the roles of CAMKKs in neurodegenerative diseases^1,10^. Furthermore, given the importance of Ca^2+^ signalling in cardiac function it is not surprising that there are important roles for CAMKKs in cardiovascular biology. For example, CAMKK1 was recently discovered to be involved in the regulation of the mesenchymal stem cell secretome^11^.

Given the partially overlapping roles of CAMKK1 and CAMKK2, the commonality of some substrate proteins (such as CAMK1 and CAMK4) and possibility of cross-regulation of expression levels it is a challenge to assign specific roles to CAMKK1 or CAMKK2. A specific inhibitor of either protein would be a valuable research tool for this purpose. So far, the only available CAMKK inhibitors are not specific for one CAMKK protein or indeed over other human kinases. For example, it has been demonstrated that STO-609^12^, the most commonly used inhibitor of CAMKKs, is able to bind to at least seven different kinases with potency similar to CAMKK1 (ERK8, MNK1, AMPK, CK2, DYRK2, DYRK3, and HIPK2)^13^. To aid the development of CAMKK1- or CAMKK2-specific inhibitors we have screened a library of kinase inhibitors against CAMKK1 and determined the X-ray crystal structure of CAMKK1 kinase domain in complex with two different inhibitors. Despite the high sequence similarity between CAMKK1 and CAMKK2 (70% over the kinase domain), comparison of the inhibitor-bound CAMKK1 structures with the existing structures of CAMKK2 reveals small but exploitable differences between the ATP-binding sites that might be used for the future design of specific inhibitors.

## Results

### Identification of kinase inhibitors that are potent inhibitors of CAMKK1

**Figure 1:**
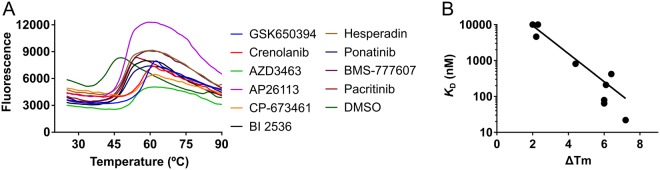
Compound screening identified various inhibitors of CAMKK1. (**A**) Thermal melting curves for CAMKK1 protein in the presence of various compounds or DMSO as a reference. (**B**) Correlation between change in melting temperature and the dissociation constant *K*_D_ for nine inhibitors where *K*_D_ values were measured by Davis *et al*.^14^. The ΔT_m_ and *K*_D_ values for these nine inhibitors can be found in the Supplementary Information.

Highly pure CAMKK1 kinase domain protein (residues Gln124-Lys411, hereafter called CAMKK1-KD) was successfully produced using an *E. coli* expression system. This protein was used to screen a library of 378 kinase inhibitors, which identified 86 molecules that bound to CAMKK1 causing a change in protein melting temperature (ΔT_m_) of greater than 2 °C (see full list in Supplementary Information). Previously, two large scale kinase screening efforts published by Davis *et al*.^14^ and Anastassiadis *et al*.^15^ included 9 and 6 (respectively) out of the 86 inhibitors identified here. Although there are only 9 inhibitors in common, the dissociation constant (*K*_D_) values measured by Davis *et al*. nevertheless show a correlation (*R*^2^ = 0.52) with the ΔT_m_ values (Fig. 1A) allowing us to estimate *K*_D_ for all of the inhibitors identified in our assay. Approximately, a ΔT_m_ of >7 °C corresponds to a *K*_D_ < 100 nM. Binding curves for inhibitors with ΔT_m_ of >7 °C are shown in Fig. 1B, and the top 20 most potent inhibitors of CAMKK1 are shown in Table 1.

**Table 1:** Top hits from DSF screening of a kinase inhibitor library against CAMKK1. ΔT_m_ values are shown as a mean and standard error from triplicate measurements. All data can be found in the Supplemental Information. Where percentage remaining activity or *K*_D_ values are available in the screening data published by Anastassiadis *et al*.^15^ or Davis *et al*.^14^ they are included in the table. Additional activity or *K*_D_ values are found in the Supplemental Information.

Compound	ΔT_m_ (°C)	Remaining activity (%)^a^		*K*_D_ (nM)^b^	
	CAMKK1	CAMKK1	CAMKK2	CAMKK1	CAMKK2
Staurosporine	23.7 ± 1.9				
GSK650394	16.1 ± 0.8				
CP-673451	14.4 ± 0.9				
Crenolanib (CP-868596)	13.6 ± 0.6				
AZD3463	11.5 ± 0.3				
BI 2536	8.8 ± 1.3				
AP26113	8.2 ± 0.6				
Volasertib (BI 6727)	7.7 ± 0.8				
BMS-777607	7.5 ± 0.3				
Sunitinib Malate	7.4 ± 0.9			22	23
Hesperadin	7 ± 0.8				
KW-2449	6.4 ± 0.3				
Pacritinib (SB1518)	5.7 ± 1.2	52	65	420	1500
Ponatinib (AP24534)	4.4 ± 3.1				
Tivozanib (AV-951)	2.6 ± 3.1			210	470

^a^From Anastassiadis *et al*.^15^ where inhibitors were screened at 1.0 µM.

^b^From Davis *et al*.^14^.

Among the compounds identified as potent inhibitors of CAMKK1 are the ALK/IGF1R inhibitor AZD3463, the PDGFR inhibitors CP-673451, the PDGFR/FLT3 inhibitor Crenolanib, and the SGK inhibitor GSK650394^16^ (which we previously also identified as a CAMKK2 inhibitor). The range of different chemotypes identified as potent CAMKK1 inhibitors suggests CAMKK1 as a common off-target of existing kinase inhibitors, and that development of CAMKK1-specific inhibitors will require a design that incorporates CAMKK1-specific structural features.

### CAMKK1 has a similar overall structure to CAMKK2

Crystallisation trials of CAMKK1-KD with a selection of the strongest binding inhibitors from the DSF analysis yielded crystals in complex with Crenolanib, AZD3463, AP26113, hesperadin and GSK650394. Crystals were also obtained with STO-609 which is well-known as a CAMKK inhibitor^10,12^. However, crystals that diffracted X-rays to high resolution were only obtained with hesperadin and GSK650394. From these crystals it was possible to determine the crystal structure of CAMKK1 to 2.1 Å resolution in complex with hesperadin and 2.2 Å resolution in complex with GSK650394 (Table 2). The two crystal structures were in different crystal forms, with two or four CAMKK1 molecules in the asymmetric unit. The final model for the co-crystal structure with hesperadin consists of residues 124–163 and 193–411, one molecule of hesperadin per CAMKK1 monomer and 271 water molecules. The disordered residues from 164–192 that are not visible in the structure are the RP-insert domain, which is implicated in substrate recognition. This RP-insert domain was also disordered in the co-crystal structure with GSK650394.

**Table 2:** Data collection and refinement statistics.

Ligand	Hesperadin	GSK650394
PDB ID	6CCF	6CD6
**Data collection**		
X-ray source	APS 24-ID-E	APS 24-ID-E
Wavelength (Å)	0.97918	0.97918
Space group	*P*2_1_	*P*2_1_
Cell dimensions *a, b, c* (Å); β (°)	49.2, 83.2, 80.1; 97.3	44.4, 96.0, 141.3; 90.03
Molecules/asymmetric unit	2	4
Resolution (Å)*	19.87-2.10 (2.16-2.10)	19.94-2.20 (2.26-2.20)
No. of unique reflections*	37,282 (3,072)	59,443 (4,575)
*R*_merge_ (%)*	11.1 (98.7)	8.30 (127.8)
Mean I/σ(I)*	9.8 (1.7)	12.1 (1.5)
Mean CC(1/2)*	0.999 (0.893)	1.000 (0.500)
Completeness (%)*	99.5 (99.8)	98.8 (98.3)
Redundancy*	5.2 (4.7)	7.1 (7.4)
**Refinement**		
Resolution (Å)	19.87-2.10 (2.16-2.10)	19.94-2.20 (2.26-2.20)
*R*_cryst_/*R*_free_ (%)	21.1/24.5	23.4/28.5
No. of atoms (protein/solvent/ligand)	4295/272/81	8323/110/116
Mean *B*-factors (protein/solvent/ligand) (Å^2^)	35/38/39	62/49/45
R.m.s.d. bond lengths (Å), angles (°)	0.008, 1.28	0.009, 1.25
**Ramachandran statistics (%)**		
Favored/Allowed/Outliers	97.3/2.7/0	96/3.9/0.1

^*^Values in parentheses represent the highest resolution shell.

**Figure 2:**
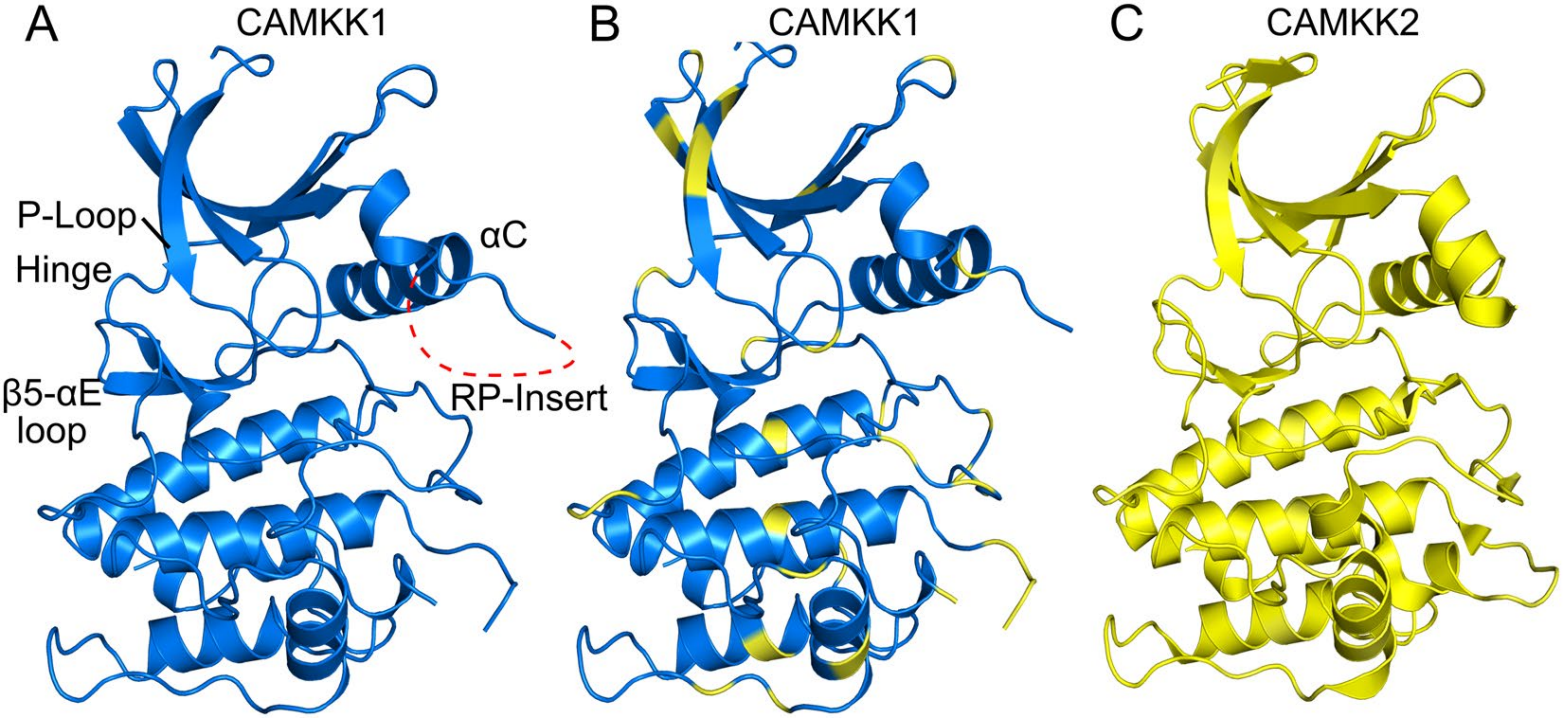
Overall structure of CAMKK1 and comparison to CAMKK2. (**A**) CAMKK1 structure. Relevant structural features described in the text are annotated. (**B**) CAMKK1 coloured in blue, but with residues not-conserved in CAMKK2 coloured in yellow. (**C**) CAMKK2 structure in complex with GSK650394 (PDB ID 6BKU). The structures of CAMKK1 and CAMKK2 with GSK650394 can be superimposed with a r.m.s.d. of 0.66 over 261 Cα atoms.

Overall, the CAMKK1 kinase domain has the expected protein kinase fold with an N-terminal lobe mostly of β-strands connected by a flexible linker (hinge region) to a predominantly α-helical C-terminal lobe (Fig. 2). In both the structures, the inhibitors hesperadin and GSK650394 are bound to the ATP-binding pocket located between the N-lobe and C-lobe. As expected from the relatively high sequence identity to CAMKK2 (70% over the kinase domain), the CAMKK1 kinase domain structure can be superimposed with the CAMKK2 kinase domain structure (PDB ID 6BKU) with a low r.m.s.d. of 0.66 Å over 261 Cα atoms.

CAMK1 and CAMK4 have an αD helix (e.g. ^108^LFDRIVEKGF^117^ in CAMK1D, PDB: 2JC6) which is involved in a hydrophobic interaction with the calmodulin binding domain during activation of CAMK1 or CAMK4 by Ca^2+^/CaM^10^. This helix may also be involved with binding the RP-insert of CAMKKs during activation of the CAMKs^10^. The positively charged RP-insert (situated between β3 and αC) from CAMKKs is unnecessary for autophosphorylation, but necessary for selective recognition of the negatively charged αD from CAMK1 and CAMK4^17^. In CAMKK1 (as well as CAMKK2) there is no αD helix (Fig. 2); it is replaced by a loop with one proline that is conserved in the CAMKKs (CAMKK1: ^235^KGPVMEVPCDKPF^247^, CAMKK2: ^272^QGPVMEVPTLKPL^284^). Therefore the mechanisms by which CAMKKs interact with calmodulin for activation might be different compared to the mechanism of CAMK1 activation^10^. The calmodulin binding domain in CAMKK1 is at the C-terminus of the protein, comprising the residues ^438^VRLIPSWTTVILVKSMLRK^456^, immediately after the auto-inhibitory domain^18^, and was not included in the construct used for crystallisation.

### Analysis of inhibitor binding to CAMKK1

**Figure 3:**
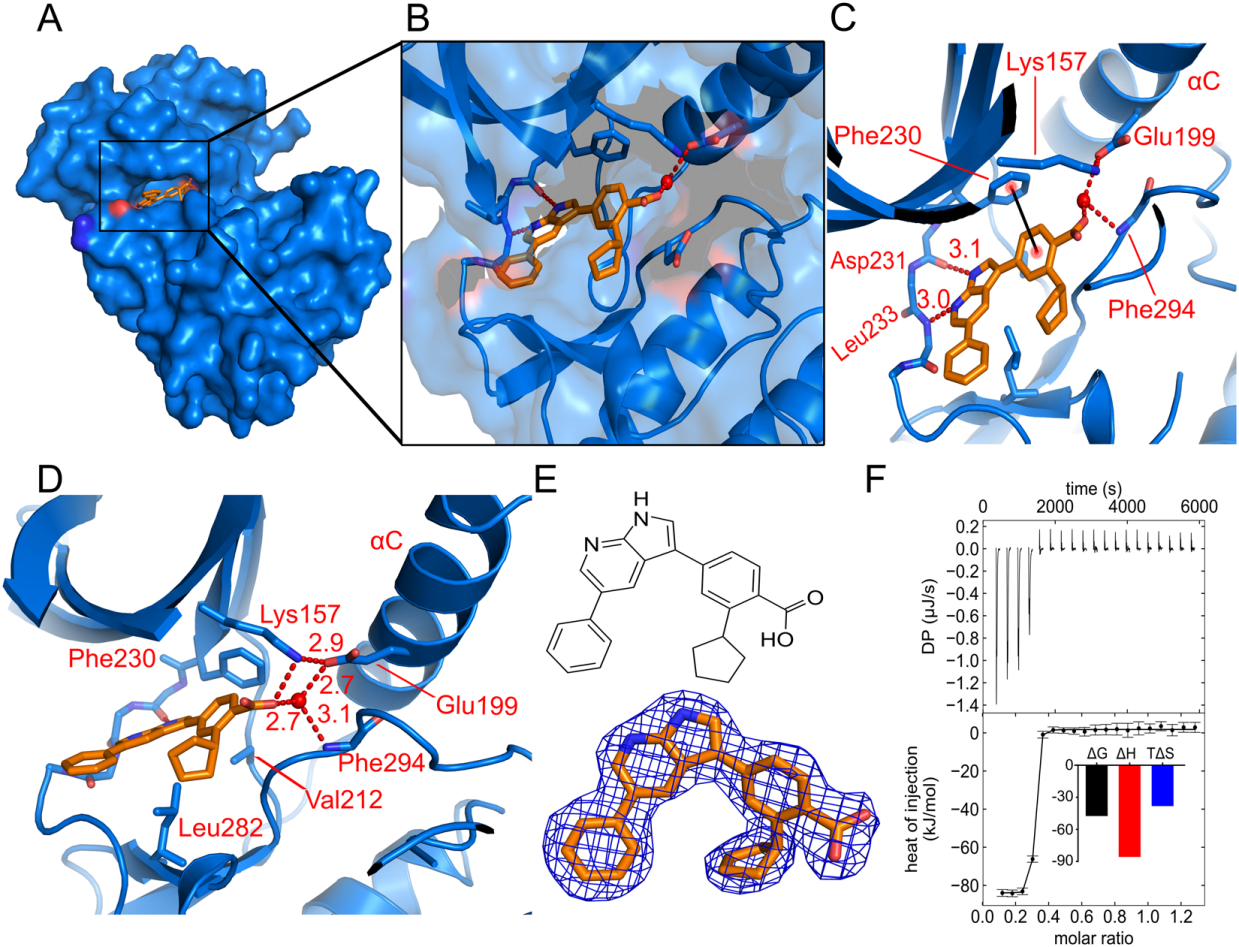
The binding of inhibitor GSK650394 to CAMKK1. (**A**,**B**) GSK650394 (shown in orange) binds in the ATP-binding pocket of CAMKK1 (shown in blue). (**C**) GSK650394 binds to the hinge region of CAMKK1 via two hydrogen bonds from its pyrrolo[2,3-b]pyridine moiety to Asp231 and Leu233, as well as a π-stacking interaction between the Phe230 and the benzoic acid moiety. (**D**) A water molecule bridges among Glu199, Phe294 and the carboxylic acid group from GSK650394. (**E**) Chemical structure of GSK650394, and a 2*F*_o_ − *F*_c_ electron density map contoured at 1.0σ for GSK650394 in the crystal structure. (**F**) ITC measured a *K*_D_ of 4 nM for the interaction, which had favourable enthalpy and unfavourable entropy of binding. Values for binding energies are shown in kJ/mol.

**Figure 4:**
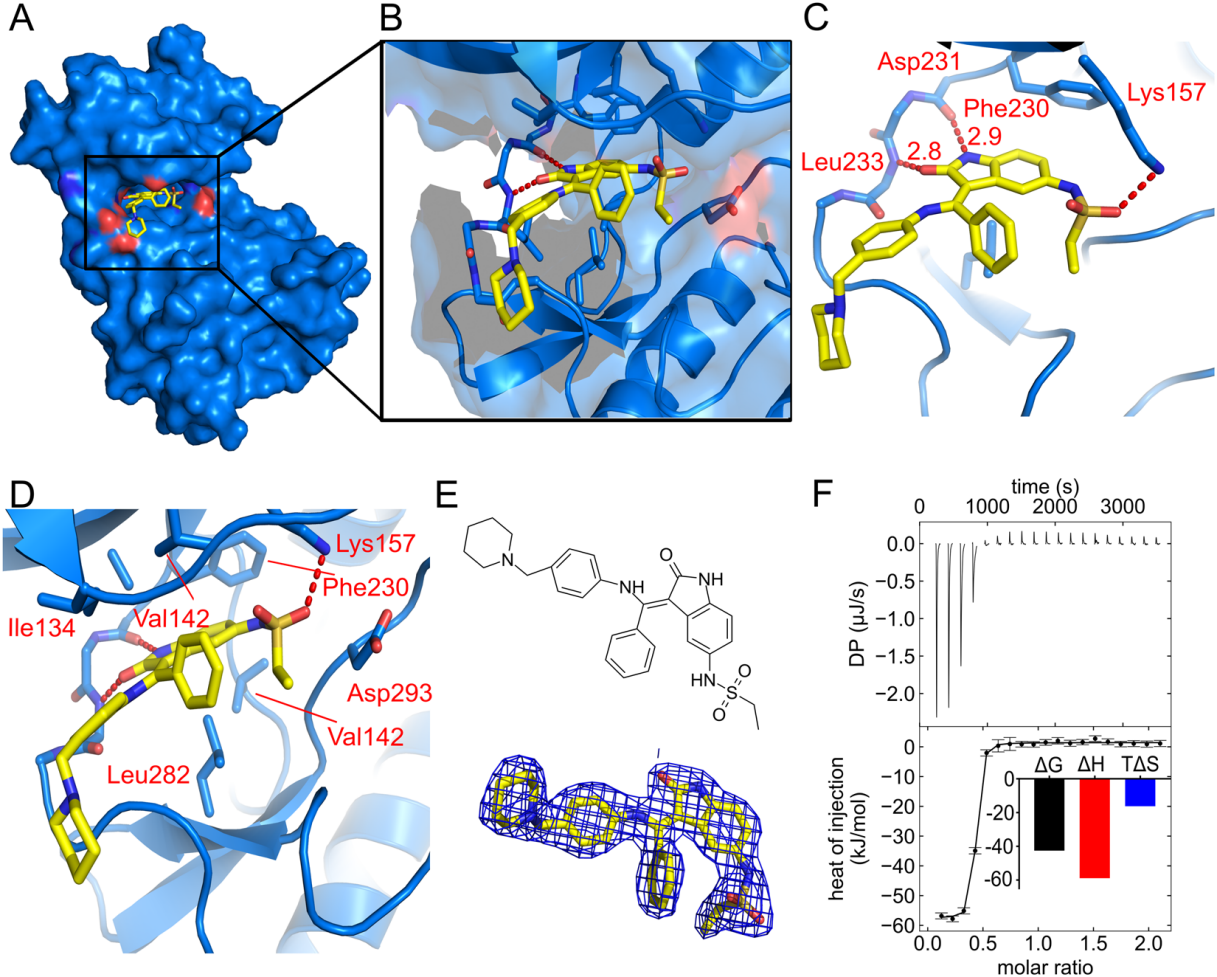
The binding of inhibitor hesperadin to CAMKK1. (**A**,**B**) Hesperadin (shown in yellow) binds in the ATP-binding pocket of CAMKK1 (shown in blue). (**C**) Hesperadin binds to the hinge region of CAMKK1 via two hydrogen bonds from its oxindole moiety to Asp231 and Leu233. (**D**) A hydrogen bond between a sulfonamide oxygen of hesperadin and Lys157 contributes to the binding energy. (**E**) Chemical structure of hesperadin, and 2*F*_o_ − *F*_c_ electron density map contoured at 1.0σ for hesperadin in the crystal structure. (**F**) ITC measured a *K*_D_ of 26 nM for the interaction, which had favourable enthalpy and unfavourable entropy of binding. Values for binding energies are shown in kJ/mol.

In both crystal structures the inhibitors were clearly visible in the electron density and could be convincingly modelled (Figs 3 and 4). The inhibitors bind as expected in the ATP-binding pocket, between the N-terminal and C-terminal lobes of the kinase domain. In the structure with GSK650394 the inhibitor is bound in a similar manner to that seen in CAMKK2 (PDB ID 6BKU), with two hydrogen bonds to the kinase hinge region, one to the backbone carbonyl atom of Asp231 and the other to the backbone nitrogen atom of Leu233 (Fig. 3C). There is a favourable aromatic π-π interaction between the benzoic acid ring of GSK650394 and the side-chain of Phe230, and a hydrophobic interaction between the phenyl ring of GSK650394 and Pro237 on the hinge. The carboxylic acid of GSK650394 forms a hydrogen bond with the side-chain of Lys157, for a total of three hydrogen bonds in direct interaction with CAMKK1. There is also a hydrogen bond to a water molecule which is bound by the side-chain of the αC residue Glu199 and the backbone nitrogen of Phe294 (Fig. 3D). ITC measurements showed that CAMKK1 binds GSK650394 strongly, with a *K*_D_ of 4 nM (Fig. 3F).

In the structure with hesperadin, the inhibitor again forms two hydrogen bonds to the hinge, via its oxindole moiety to the backbone carbonyl atom of Asp231 and the backbone nitrogen atom of Leu233 (Fig. 4C). Hesperadin also forms a hydrogen bond via its sulphonamide to the side-chain of Lys157, while the oxindole ring forms a hydrophobic interaction with Phe230 (Fig. 4C,D). ITC measurements showed that CAMKK1 binds hesperadin with a *K*_D_ of 26 nM (Fig. 4F).

### The most potent CAMKK1 inhibitors bind due to favourable enthalpy

**Figure 5:**
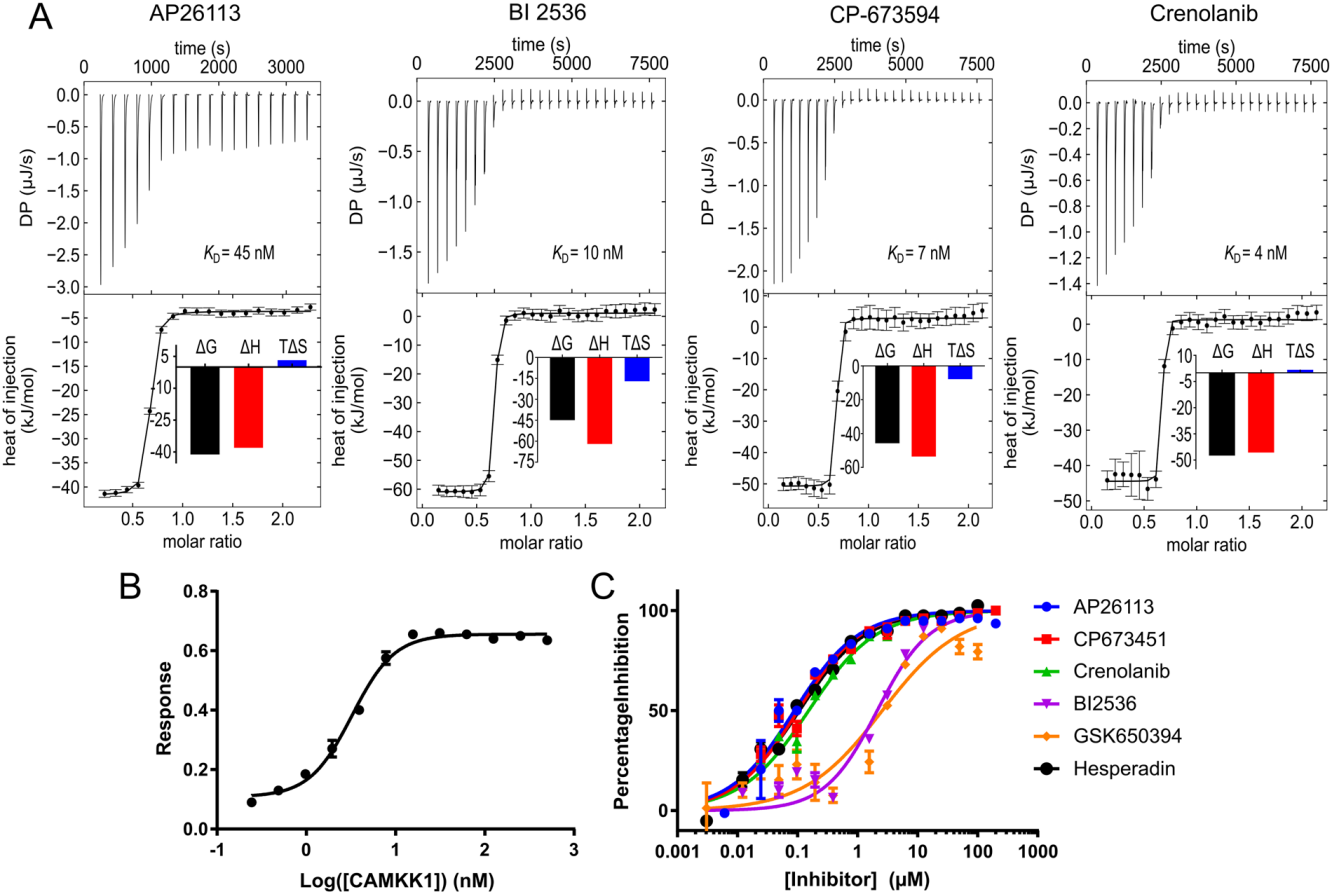
(**A**) Dissociation constant measurements by ITC for four additional inhibitors that promoted a change in the melting temperature (ΔT_m_) of CAMKK1 by more than 7 °C. The top part of each figure shows the injection heats, the bottom part shows the fitted binding isotherms (a single-site model was used), and the insets show the binding energies in kJ/mol. (**B**) CAMKK1-FL showed strong enzymatic activity on a peptide substrate in the presence of Ca^2+^/calmodulin. A final concentration of 5 nM CAMKK1-FL was chosen for use in the IC_50_ determinations. (**C**) IC_50_ determinations of selected inhibitors. Two compounds behaved poorly in the assay, possibly due to solubility problems. Calculated IC_50_ values are shown in Table 3.

After seeing that hesperadin and GSK650394 were extremely potent inhibitors of CAMKK1, we measured ITC data for four additional inhibitors that bound strongly to CAMKK1 in the DSF assay (Fig. 5, Table 3), to confirm if the low dissociation constants and enthalpy-dependent binding seen with hesperadin and GSK650394 were common features of the identified CAMKK1 inhibitors. The data show that all of the tested inhibitors are extremely potent binders of CAMKK1, and furthermore in all cases the binding is heavily enthalpy-dependent. Finally, to confirm that the compounds with the strongest binding to CAMKK1 are indeed inhibitors of its enzymatic activity we prepared full length CAMKK1 protein (CAMKK1-FL) and performed an IC_50_ determination by enzymatic assay using CAMKK1-FL in the presence of Ca^2+^/calmodulin (Table 3 and Fig. 5B,C).

**Table 3:** Binding affinities for selected inhibitors measured by ITC at 20 °C and IC_50_ values for inhibition of enzymatic activity, showing the good correlation of *K*_D_ with thermal stabilisation of CAMKK1 measured by DSF.

Inhibitor	*K*_D_ (nM)	ΔH (kJ/mol)	Fraction incompetent A (ligand)	Fraction incompetent B (protein)	ΔT_m_ (°C)	IC_50_ (nM)
GSK650394	4	−85.8	0.7	0.0	16.1 ± 0.8	25800(*)
Crenolanib	4	−45.6	0.4	0.0	13.6 ± 0.6	153
CP-673451	7	−53,5	0.4	0.0	14.4 ± 0.9	101
BI 2536	10	−61.9	0.4	0.0	8.8 ± 1.3	2120(*)
Hesperadin	26	−58.9	0.6	0.0	7 ± 0.8	107
AP26113	45	−38.1	0.4	0.0	8.2 ± 0.6	89

ΔT_m_ values are the same as in Table 2. IC_50_ values were determined at an ATP concentration of 100 µM. (*) Potentially outliers due to poor solubility in the assay.

### Two active site differences could allow the design of specific inhibitors

**Figure 6:**
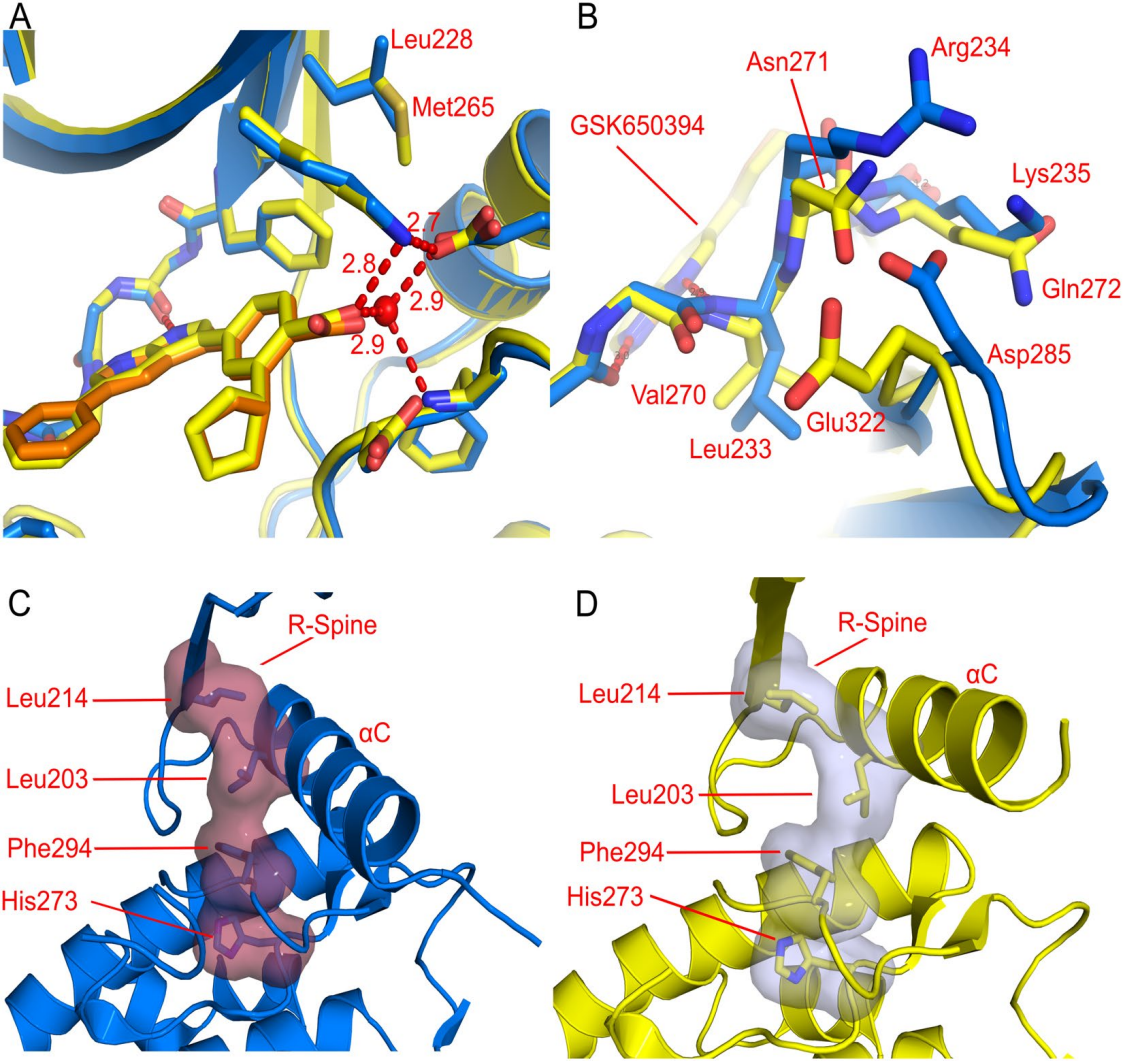
Structural differences between CAMKK1 (shown in blue) and CAMKK2 (shown in yellow) that could be exploited to design specific inhibitors. (**A**) Adjacent to the kinase R-spine Leu228 in CAMKK1 is Met265 in CAMKK2. An inhibitor that disrupts the R-spine (a type 2 inhibitor) could engage this amino acid. (**B**) Due to different interactions between the hinge region (Arg234 in CAMKK1) and the β4-β5 loop (Asp285 in CAMKK1) the hinge region is significantly further away from the ATP-binding site in CAMKK2 (1.2 Å). This could be exploited by an inhibitor that interacts with the backbone of Arg234 (or Asp285 for a CAMKK2 inhibitor). (**C**) View of the R-spine residues and αC helix of CAMKK1 when bound to GSK650394. (**D**) View from the same angle as (**C**) of the R-spine residues and αC helix of CAMKK1 when bound to hesperadin, showing the deformation of the R-spine and the more outward position of αC in the presence of this inhibitor, and the flexibility of these residues.

The ATP-binding sites of CAMKK1 and CAMKK2 are highly conserved, which is reflected in the similarity of the inhibitor binding profiles of CAMKK1 and CAMKK2 seen previously^14,15^ Comparison of the CAMKK1 structures to CAMKK2 reveals two possible options for the design of specific inhibitors (Fig. 6). The first is that there are differences in the hydrophobic “back-pocket” region around the kinase regulatory spine (R-spine). In particular, Leu228 in CAMKK1 is Met265 in CAMKK2 and this difference would presumably affect the movement of the R-spine residues in response to inhibitors that disrupt the R-spine, i.e. type II kinase inhibitors. A type II inhibitor would have the potential to gain selectivity between CAMKK1 and CAMKK2 by taking advantage of this and other non-conserved residues outside the ATP-binding pocket itself.

However, a more significant difference between CAMKK1 and CAMKK2 is seen in the kinase hinge region (Fig. 6B). The hinge residues Leu233, Arg234 and Lys235 are replaced in CAMKK2 by Val270, Asn271 and Gln272. Although none of these amino acid side-chains are directed into the ATP-binding site, these sequence differences cause a significant difference in the conformation of the protein backbone in this region, greater than 1 Å difference in position. Especially important is CAMKK1 Leu233, which is larger than the equivalent valine residue in CAMKK2 and to be accommodated in the same space requires the different backbone conformation observed. This difference may be the reason for the previously observed differences in binding to the inhibitor STO-609^10^. The differences in the hinge residues Arg234 and Lys235 which interact with the β6-β7 loop are also likely to contribute to the different conformation, especially since the key residue on the β6-β7 loop, Asp285, is also not conserved in CAMKK2.

## Discussion

The compound screening data showed that a significant number of kinase inhibitors interact with CAMKK1, despite none of these molecules being originally designed as a CAMKK inhibitor. This suggests that CAMKK1 is an easily inhibited kinase, and given the strength of binding observed, that alteration of Ca^2+^/CaM signalling may be a significant cause of some of the phenotypes observed with some of these inhibitors. It also suggests that achieving high potency with a newly designed CAMKK1-specific inhibitor will be possible, especially given the relatively high *K*_M_(ATP) previously reported for CAMKK1^19,20^.

As the ATP-binding sites of CAMKK1 and CAMKK2 are so similar, for future design of a specific inhibitor there are two possibilities: an allosteric inhibitor, or an inhibitor which can take advantage of the small differences that do exist to achieve specificity. The two different crystal structures of CAMKK1 with different inhibitors show a large difference in the position of helix αC (Fig. 6C,D). In the structure with hesperadin αC is in an inactive αC-out conformation, with a disrupted kinase R-spine (Fig. 6D). The R-spine is a set of four residues in kinases that line up to form a hydrophobic spine when a protein kinase is in an active conformation^21^. Since it appears that hesperadin does not directly push out the αC helix, this indicates flexibility of this region of the protein and therefore that a type II inhibitor (or indeed an allosteric, type III, inhibitor) binding to and disrupting the R-spine would be possible. The inhibitor screening data shows that a number of known type II inhibitors such as ponatinib bind strongly to CAMKK1. Given that there are sequence differences between CAMKK1 and CAMKK2 in this region of the R-spine and αC, this could be a way to achieve selectivity. Finally, the significant differences in the conformation of the C-terminal part of the kinase hinge region (residues Arg234-Gly236, Fig. 6B) could be exploited by suitably-designed type I kinase inhibitors.

## Materials and Methods

### DNA constructs

A full length coding DNA clone for human CAMKK1 was obtained from the Mammalian Gene Collection (IMAGE Consortium Clone ID 5751570, corresponding to CAMKK1 isoform A, Uniprot Q8N5S9-1) and used as template for PCR amplification. The kinase domain (Gln124 to Lys411) was amplified using the primers: forward TACTTCCAATCCATGCAGCTGAACCAGTACAAGCTG, reverse TATCCACCTTTACTGTCACTTGGTCACCCAAGGATGCAACTTGATGTC, and cloned into vector pNIC28-Bsa4. The full length sequence (Met1 to Ser505) was amplified using the following primers: TACTTCCAATCCATGGAGGGGGGTCCAGCTGT (forward) and TATCCACCTTTACTGCTGGATGCAGCCTCGTCTTC (reverse), and cloned into baculovirus transfer vector pFB-Bio5. The constructs were cloned using ligation-independent cloning^22^ and were verified by DNA sequencing.

### Protein expression and purification

CAMKK1-Q124-K411 (CAMKK1-KD) in pNIC28-Bsa4 was transformed into chemically competent *Escherichia coli* BL21(DE3)-R3 cells that co-express λ-phosphatase and three rare tRNAs (plasmid pACYC-LIC+). Colonies were used to inoculate 50 mL of LB media supplemented with 5 mM betaine, 50 µg/mL kanamycin and 35 µg/mL chloramphenicol which was left shaking at 37 °C overnight. This culture was used to inoculate 1.5 L of Terrific Broth containing 50 µg/mL kanamycin at 37 °C until the OD_600_ reached 3. The culture was cooled to 18 °C, 0.2 mM of Isopropyl β-D-1-thiogalactopyranoside (IPTG) was added, and left overnight. Cells were collected by centrifugation for 15 min at 7,500 × g at room temperature. The cell pellet was weighed and resuspended in the same volume of 2 × binding buffer (1 × binding buffer was 50 mM HEPES pH 7.5, 500 mM NaCl, 10% glycerol, 10 mM imidazole, 1 mM tris(2-carboxyethyl)phosphine (TCEP) with protease inhibitors set II (Calbiochem) at 1:200 ratio) and frozen at −80 °C until use.

For purification, the cell pellet was thawed and sonicated on ice. Polyethyleneimine (pH 7.5) was added to the lysate to a final concentration of 0.15% (w/v) and the sample was centrifuged at 53,000 × g for 45 min at 4 °C. The supernatant was loaded onto an immobilised metal affinity chromatography (IMAC) column (5 mL HisTrap FF Crude) and washed in binding buffer containing 30 mM imidazole. The protein was eluted with elution buffer (binding buffer with 300 mM imidazole). To remove the 6xHis-tag, the protein was incubated with TEV protease during dialysis overnight at 4 °C into gel filtration buffer (20 mM HEPES, 500 mM NaCl, 5% glycerol, 1 mM TCEP, 5% [v/v] glycerol). Protein was further purified using reverse affinity chromatography on Ni-Sepharose followed by gel filtration (Superdex 200 16/60, GE Healthcare). Protein in gel filtration buffer was concentrated to 10.4 mg/mL (measured by UV absorbance with a NanoDrop spectrophotometer (Thermo Scientific) using the calculated molecular weight and estimated extinction coefficient) using 10 kDa molecular weight cut-off centrifugal concentrators (Millipore) at 4 °C.

Full length CAMKK1 (CAMKK1-FL) protein was expressed in *Spodoptera frugiperda* insect cells (*Sf9*), using the Bac-to-Bac® Baculovirus expression system (Invitrogen), following the procedures previously described by Mahajan *et al*.^23^. Following P2 baculovirus preparation, 3 × 500 mL volumes of *Sf9* cells at a density of 2.2 × 10^6^ cells/mL were infected with baculovirus and incubated at 27 °C with shaking for 72 h.

For purification, the cells were resuspended in 50 mM Tris·HCl, pH 8.0, 300 mM NaCl, 10 mM imidazole, 10% glycerol and 1 mM TCEP. The protein was purified on an IMAC column as for CAMKK1-KD, and eluted in 50 mM Tris·HCl, pH 8.0, 300 mM NaCl, 300 mM imidazole, 10% glycerol and 1 mM TCEP. The eluted protein was dialysed into 20 mM Tris·HCl, pH 8.0, 150 mM NaCl, 20% glycerol and 1 mM TCEP and frozen at −80 °C. Aliquots were thawed and used for the enzymatic assays.

### Differential scanning fluorimetry

CAMKK1-KD protein was screened against a library of 378 structurally diverse and cell-permeable ATP-competitive kinase inhibitors available from Selleckchem (Houston, TX, United States; catalog No. L1200). DSF measurements were made in a 384-well plate. Each well contained 20 μL of 1 μM kinase in 100 mM potassium phosphate pH 7.0, 150 mM NaCl, 10% glycerol and the Applied Biosystems Protein Thermal Shift dye at the recommended concentration of 1:1000.

The compounds, previously solubilized in DMSO, were used at 10 µM final concentration and 0.1% DMSO. Plates were sealed using optically clear films and transferred to a QuantStudio 6 qPCR instrument (Applied Biosystems). The fluorescence intensity was measured during a temperature gradient from 25 to 95 °C at a constant rate of 0.05 °C/s and protein melting temperatures were calculated based on a Boltzmann function fitting to experimental data, as implemented in the Protein Thermal Shift Software (Applied Biosystems). Protein in 0.1% DMSO was used as a reference. Compounds that caused a shift in melting temperature of the protein (ΔT_m_) of 2 °C or higher compared to the reference were considered positive hits.

### IC_50_ Determination

Enzymatic activity was measured using the full length CAMKK1 protein and a biotinylated peptide bound to streptavidin-XL665, with detection by a phosphor-specific antibody conjugated to Eu-cryptate (Cisbio Kinease kit, #62ST1PEB); an increase in phosphorylation results in increase TR-FRET signal. Assays were performed in black 384-well plates (Corning #4514), in 10 µL volumes in enzymatic assay buffer (Cisbio) composed of 4 µL of 2.5x inhibitor, 2 µL of 5x enzyme mix and 4 µL of 2.5x ATP and peptide. Final assay concentrations of the components of the enzyme mix were 5 nM CAMKK1-FL, 1 µM calmodulin, 1 mM CaCl_2_, 5 mM MgCl_2_, 1 mM DTT. Final concentrations of ATP and peptide were 100 µM ATP, 1 µM peptide (STK substrate S1). The inhibitor solutions were prepared starting from 10 mM solutions in DMSO, diluted in enzymatic assay buffer to 200 or 100 µM, and then a 2x serial dilution in enzymatic assay buffer was performed, yielding the serially diluted inhibitors at 2.5x final concentration. After addition of the inhibitor and enzyme mix, the assay plate was incubated for 1 hour at 4 °C, the ATP and peptide solution was added, and the plate incubated for 2.5 hours at 28 °C. For detection, 5 µL of streptavidin-XL665, and 5 µL of antibody-Eu-cryptate were added. TR-FRET measurements were made using a CLARIOstar plate reader (BMG Labtech) with excitation at 330 nm, emission at 665 nm and 620 nm, integration delay 60 µs and integration time 400 µs. Data was analysed in GraphPad PRISM version 7.

### Crystallisation, data collection and structure determination

For crystallization, inhibitors previously identified were added to purified CAMKK1-KD at a threefold molar excess and the samples were incubated on ice for one hour. Initially, 40 μL of purified protein at 10.4 mg/mL was diluted in 360 μL of gel filtration buffer, passed to a fresh 1.5 mL tube containing 5.8 μL of the inhibitor (10 mM in DMSO), and incubated for one hour. The mixture was concentrated at 4 °C in an Amicon Ultra 10 kDa MW cutoff concentrator (Millipore) for DMSO removal. Twice, 300 μL of gel filtration buffer was added and the sample concentrated again to 40 μL final volume. Crystallization was performed in SWISSCI 3-well low profile crystallization plates at 6 °C. Crystals of CAMKK1-KD with hesperadin or GSK650394 grew from a mixture of 100 nL of protein:inhibitor solution and 100 nL of a reservoir solution of 33% PEG2000 MME, 0.1 M potassium thiocyanate, CHC buffer (0.1 M each citric acid, HEPES, and CHES) pH 7.5. Crystals were cryo-protected in reservoir solution supplemented with 30% ethylene glycol before flash-freezing in liquid nitrogen for data collection (Table 1).

The data was integrated with XDS^24^ and scaled using AIMLESS from the CCP4 software suite^25^. The structure was solved by molecular replacement using Phaser^26^ and the kinase domain of CAMKK2 as the search model (PDB ID 2ZV2)^10^. Refinement was performed using REFMAC5^27^ and Coot^28^ was used for model building. Structure validation was performed using MolProbity^29^.

### Isothermal titration calorimetry

Measurements were made using a MicroCal AutoiITC200 (Malvern, United Kingdom), at 20 °C with 1000 rpm stirring. For all measurements CAMKK1-KD protein was dialysed overnight against gel filtration buffer, and the dialysis buffer was used to dilute the inhibitors. CAMKK1-KD protein was titrated into a solution containing the inhibitor. The concentrations used for each measurement were: hesperadin 27 µM, CAMKK1-KD 270 µM (measurement made using 2 µL injections and 180 s between each injection); AP26113, BI2536, Crenolanib or CP-673451 26.7 µM, CAMKK1-KD 267 µM (measurements made using 1.5 µL injections and 300 s between each injection); GSK650394 30.8 µM, CAMKK1-KD 247 µM (measurement made using 1.5 µL injections and 300 s spacing between each sample). ITC data was analysed with NITPIC, and SEDPHAT; figures were made using GUSSI^30^.

## Electronic supplementary material

DSF screening data

## Data Availability

The coordinates and structure factors for the crystal structures of CAMKK1 have been deposited in the Protein Data Bank with accession codes 6CCF and 6CD6.
